# Significance of Early Postoperative Arterial Lactic Acid, Inferior Vena Cava Variability, and Central Venous Pressure in Hypovolemic Shock

**DOI:** 10.1155/2019/6504916

**Published:** 2019-11-11

**Authors:** Wei Lin, Xingsheng Lin, Yingfeng Zhuang, Xiaobin Pan, Chao Wu, Shujuan Zhang, Lihui Zhang, Jian Lin, Songjing Shi, Songchang Shi

**Affiliations:** ^1^Department of Endocrinology, Shengli Clinical Medical College of Fujian Medical University, Fujian Provincial Hospital, Fuzhou 350001, China; ^2^Department of Critical Care Medicine, Shengli Clinical Medical College of Fujian Medical University, Fujian Provincial Hospital South Branch, Fuzhou 350001, China; ^3^Department of Critical Care Medicine, Shengli Clinical Medical College of Fujian Medical University, Fujian Provincial Hospital, Fuzhou 350001, China

## Abstract

**Introduction:**

Up to one-third of patients admitted to the ICU are in circulatory shock, and early recognition of the condition is vital if subsequent tissue injuries are to be avoided. We would like to know what role the arterial lactic acid, inferior vena cava variability, and CVP (central venous pressure) play in the early stages of shock.

**Methods:**

This is a retrospective study of patients who underwent surgical resuscitation in the Department of Critical Care Medicine. We use the ROC (receiver-operating characteristic) curve to evaluate the significance of each indicator in the diagnosis. For correlation analysis between groups, we first use linear regression for processing and then analysis with correlation.

**Results:**

The ROC curve analysis shows that the area under the curve of the lactic acid group was 0.9272, the area under the curve of the inferior vena cava variability group was 0.8652, and the area under the curve of the CVP group was 0.633. Correlation analysis shows that the inferior vena cava variability and arterial lactic acid Pearson's *r* = 0.2863 and CVP and arterial lactic acid Pearson's *r* = 0.0729.

**Conclusion:**

The diagnostic value of arterial lactate is still very high and can still be used as an early warning indicator to help clinicians be alert to the microcirculatory disorders that have emerged quietly. The degree of inferior vena cava variability is linearly related to arterial lactic acid and can also be used as a reference indicator for early evaluation of shock. The diagnostic value of CVP is obviously lower.

## 1. Introduction

Shock is best defined as a life-threatening, generalized form of acute circulatory failure associated with inadequate oxygen utilization by the cells, including mottled skin, acrocyanosis, slow capillary refill time, and an increased central-to-toe temperature gradient [1]. Shock is a state of acute circulatory failure resulting from four mechanisms. The first of these is a decrease in venous return due to a loss of circulating volume. The second is a failure of the pump function of the heart that results from a loss of contractility or a major arrhythmia. The third is an obstruction due to pulmonary embolism, tension pneumothorax, or cardiac tamponade. The fourth is the loss of vascular tone that results in maldistribution of blood flow [2]. Up to one-third of patients admitted to the ICU are in circulatory shock, and early recognition of the condition is vital if subsequent tissue injuries are to be avoided [3].

Traditionally, we are used to assessing shock through blood pressure, urine output, heart rate, etc. With the increasing number of evaluation methods, we also began to use lactic acid, ultrasound evaluation of the inferior vena cava diameter, central venous pressure (CVP), and so on. Because these indicators are very easy to obtain, they are easy to monitor at any time. However, we would like to know what role the arterial lactic acid, inferior vena cava variability, and CVP play in the early stages of shock. This will be the focus of our research.

## 2. Methods

### 2.1. Study Design

This is a retrospective study of patients who underwent surgical resuscitation in the Department of Critical Care Medicine, from May 2015 to February 2019. This study was approved by the Human Subjects Committee of our institutional review board with a waiver from informed consent.

### 2.2. Study Subjects

The applicants were patients who underwent surgery in the hospital at the Department of Critical Care Medicine. The patients were transferred to our department under sedative muscle relaxation and continued to receive ventilator support treatment. The following measures were observed: PEEP ≤ 5 mmHg [4], tidal volume 8 ml/kg, oxygenation index >300, and R 14–16 times/min. Arterial blood gas analysis was performed one hour after transfer (GEM Premier 3500). During the half-hour to one-hour period of admission, we collected the patient's arterial lactate values, inferior vena cava variability, and central venous pressure. We performed the sonographic (SonoSite EDGE) evaluation of inferior vena cava diameters and estimated its collapsibility (the caval index) [5, 6]. We determined the central venous pressure (CVP) (measured by Philips Medizin Systeme, cmH2O) and recorded it. We connected the subclavian vein catheter to the transducer and connected it to the Philips monitor. After each zero adjustment, the monitor automatically readed the CVP value. According to the consensus on circulatory shock and hemodynamic monitoring (task force of the European Society of Intensive Care Medicine) [1], the presence or absence of shock was evaluated.

A total of 304 patients were collected. Among them, there were 150 cases of craniocerebral operation, 60 cases of laparoscopic radical gastrectomy, 50 cases of laparoscopic colon cancer radical operation, 30 cases of thyroid gland lobectomy, and 14 cases of bladder cancer after radical operation. All of the patients had not used catecholamine during the operations. The severity of the disease was assessed using the APACHE-II (Acute Physiology and Chronic Health Evaluation-II) scoring system with values between 5 and 10.

### 2.3. Exclusion Criteria

Exclusion criteria are as follows: patients <18 years of age, resuscitation after pregnancy surgery and cardiothoracic surgery, COPD, chronic heart failure, chronic renal failure, chronic pulmonary hypertension, MODS, and various causes of difficulty in offline extubation during resuscitation.

### 2.4. Data Collection

We judge whether there is shock based on the European consensus in 2014 [1]. Because it is defined as systolic blood pressure <90 mmHg, or mean arterial pressure (MAP) <65 mmHg, or a decrease ≥40 mmHg from baseline, we also included visualization through the three “windows” of the body [7]: the peripheral window (skin that is cold, clammy, and blue, pale, or discolored); the renal window (decreased urine output <0.5 mL/kg/h); and the neurologic window (altered mental characterized by obtundation, disorientation, and confusion). Finally, the procedure comprises three senior doctors of intensive care specialists, and all eligible patients are divided into shock and no shock. All bedside ultrasound measurements are done by intensive medical specialists who have worked for at least one year and physicians who have received professional bedside ultrasound training. All CVP measurements are done by intensive care specialists or nurses.

We use the ROC (receiver-operating characteristic) curve to evaluate the significance of each indicator in the diagnosis. For correlation analysis between groups, we first use linear regression for processing and then analysis with correlation. We performed data analysis using GraphPad Prism 7.0a statistical software (GraphPad Software Inc.).

## 3. Results

**Figure 1:**
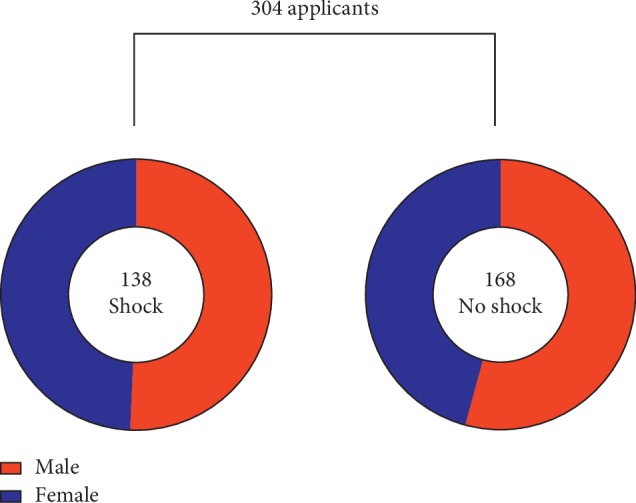
Distribution of the applicants (304 patients including 156 males, accounting for 51.3%, and 148 females, accounting for 48.7%).

A total of 304 patients included 156 males, accounting for 51.3%, and 148 females, accounting for 48.7%. The average age was 54.02 + 9.75. According to the standard, there were two groups: shock and no shock, including 138 cases with shock, accounting for 45.7%, and 168 cases without shock, accounting for 55.3% (Figure 1).

**Figure 2:**
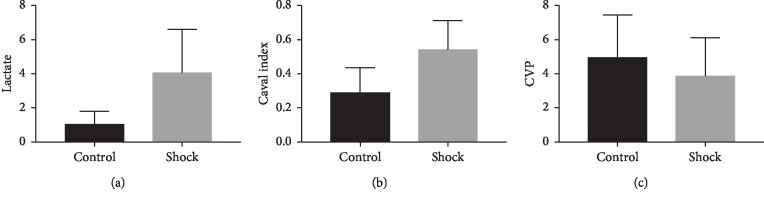
Control and shock groups.

Among the three groups of shock data, the mean value of arterial blood gas analysis in the no-shock group was 1.035 + 0.761, and the mean value of arterial blood gas analysis in the shock group was 4.051 + 2.538. The mean value of inferior vena cava variability in the no-shock group was 0.592 + 3.938, and the mean value of inferior vena cava variability in the shock group was 0.540 + 0.171. The mean CVP of the no-shock group was 4.94 + 2.490, and the average CVP of the shock group was 3.85 + 2.258 (Table 1; Figure 2).

**Figure 3:**
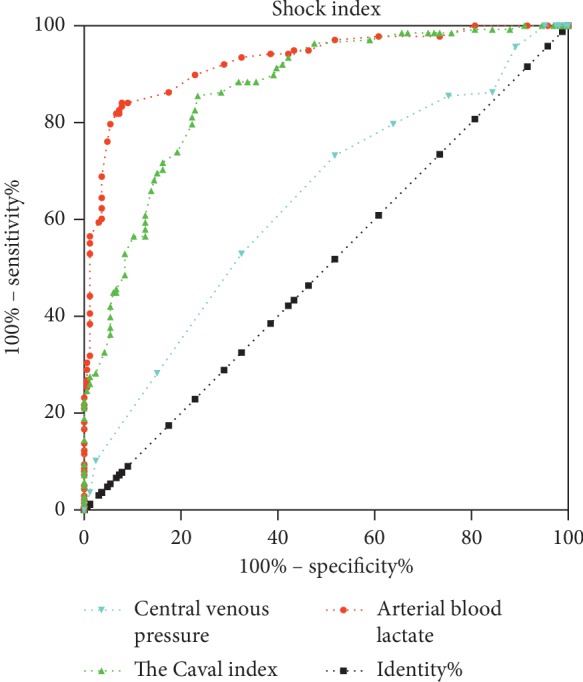
ROC curve analysis of the indexes. Red indicates the lactic acid group, for which the area under the curve is 0.9272, the corresponding standard error is 0.01534, *p* < 0.0001, and the 95% confidence interval is (0.8971, 0.9573). Green indicates the inferior vena cava variability group, for which the area under the curve is 0.8652, the corresponding standard error is 0.02042, *p* < 0.0001, and the 95% confidence interval is (0.8251, 0.9052). Blue indicates the CVP suite, for which the area under the line is 0.633, the corresponding standard error is 0.032, *p* < 0.0001, and the 95% confidence interval is (0.5702, 0.6957).

The ROC curve analysis results of the three groups of shock monitoring indicators showed that the area under the curve of the lactic acid group was 0.9272, indicating that the diagnostic value of lactic acid was better. The corresponding standard error was 0.01534, *p* < 0.0001, which was statistically significant. The 95% confidence interval was (0.8971, 0.9573). The area under the curve of the inferior vena cava variability group was 0.8652, indicating that the diagnostic accuracy was moderate, and the corresponding standard error was 0.02042, *p* < 0.0001, which was statistically significant. The 95% confidence interval was (0.8251, 0.9052). The area under the curve of the CVP group was 0.633, indicating that the diagnosis was lower, and the corresponding standard error was 0.032, *p* < 0.0001, which was statistically significant. The 95% confidence interval was (0.5702, 0.6957) (Figure 3; Table 2).

**Figure 4:**
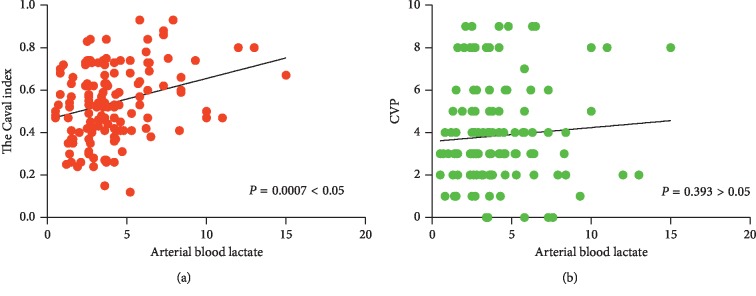
(a) Scatter plot of the inferior vena cava variability and arterial lactate (red); (b) scatter plot of CVP (central venous pressure) and arterial lactate (green).

Correlation analysis between inferior vena cava variability, CVP, and arterial lactate: inferior vena cava variability and arterial lactic acid Pearson's *r* is 0.2863, 95% confidence interval is 0.1252 to 0.4327, and *p*=0.0007. CVP and arterial lactic acid Pearson's is *r* 0.0729, 95% confidence interval is −0.09498 to 0.2375, and *p*=0.3930. Inferior vena cava had a correlation between variability and arterial lactic acid, while CVP had no significant correlation with arterial lactic acid (Figure 4; Table 3).

## 4. Discussion

This study showed that the diagnostic value of arterial blood lactate and inferior vena cava variability was good for early hypovolemic shock, and their area under the curve was as high as 0.93 and 0.87, respectively. The arterial blood lactic acid acquisition method is simple and convenient, and the clinical operation is easy, which is especially suitable for clinical work. The inferior vena cava variability has the advantages of noninvasiveness and easiness to measure repeatedly, and it has a linear correlation with arterial blood lactate (*r* = 0.30, *p* < 0.05). When arterial blood is difficult to obtain, or when repeated measurements are needed, the changes in arterial blood lactate can be determined. The central venous pressure is easily disturbed, and it has no obvious correlation with arterial blood lactic acid and cannot reflect the change of lactic acid value, so it should be used with caution.

Humans have been fighting shock, and shock has been threatening humanity [8]. For more than a decade, we have evolved from traditional tissue perfusion-monitoring indicators such as urine volume, heart rate, blood pressure, cardiac output, and pulmonary artery pressure, but the treatment of shock is still challenging. This is due to the inability to clearly monitor tissue perfusion. In the past, blood pressure was used as an important indicator of diagnosing shock. Clinicians often wait until the patient's blood pressure drops before considering shock. A random-effects model produced a sensitivity of 13% for moderate blood loss and 33% for severe blood loss. The authors therefore concluded that a systolic blood pressure <95 mmHg is not a sensitive measure for ruling out moderate or significant blood loss. A decrease in cardiac output is associated with significant vasoconstriction, leading to decreased peripheral perfusion to maintain arterial pressure [9]. It is impossible for us to routinely use invasive blood flow-monitoring methods for every patient. However, if there is no quick, easy, and minimally invasive evaluation method, it is easy to ignore the clinical manifestations of early shock, resulting in untimely treatment or even serious consequences. Therefore, how to improve the early diagnosis success rate of shock is the current challenge [10, 11].

Studies have shown that hyperlactosis is closely related to tissue hypoperfusion. When the systemic oxygen supply drops to the point where oxygen demand cannot be met, the body will have an increase in lactic acid [12, 13]. Most current research shows that resuscitation of shock targeting an incremental decrease in lactate is effective, at least during the early phase [14–16]. However, most of these studies are currently directed at septic shock. Hypovolemic shock, the most common type of shock, lacks research in this area. Unlike other shocks, early detection and effective treatment of hypovolemic shock have the opportunity to avoid adverse outcomes such as organ failure. This is also an important reason why we take this part of the patient to observe.

In our study, we consider the inferior vena cava variability in conscious patients, especially those in thoracic and abdominal surgery, which may be disturbed as the patient's breathing amplitude changes. And the research shows that PEEP below 7 mmHg has no significant effect on CVP monitoring [4]. Therefore, we selected patients who were transferred to the ICU after surgery, and who were still in the sedative muscle relaxation mode, to ensure that the effect of each respiratory movement on the pressure of the thoracic and abdominal cavity was relatively constant, and the central vein diameter and pressure were monitored. The numerical value changes [17–19].

In view of the current lack of data on early studies of hypovolemic shock, we hope to understand whether there is any difference in the diagnostic value of elevated lactic acid in the early stage of shock versus inferior vena cava variability and CVP. At the same time, we also want to know if the inferior vena cava variability and CVP can help early judgment of shock if the chest and abdomen pressure changes are eliminated. Moreover, the relevance of these three in the early stages of shock is also what we always wanted to know.

## 5. Limitations

However, this study only studies the lactic acid situation at some point in the early postoperative period. As time points change, there will be a correlation between lactic acid changes and cyclic changes. For patients with spontaneous breathing, changes in chest pressure from spontaneous breathing may also affect central venous pressure. These are the next steps that need to be analyzed.

In addition, our study design was observational and retrospective. We also hope to have a forward-looking, multicenter, big data research to obtain more objective data. These data were written by a variety of medical providers (residents, attending physicians, and nurse practitioners) with a possibility of misclassification bias. Furthermore, a physician's medical decision-making process may incorporate consensus guidelines, and different providers may have used these consensus guidelines to a variable degree based on their training.

## 6. Conclusion

Our study shows that, for patients with no significant chronic heart, lung, kidney, and other vital organ failure, the diagnostic value of arterial lactate is still very high and can still be used as an early warning indicator to help clinicians be alert to the microcirculatory disorders that have emerged quietly. Although the inferior vena cava variability is affected by a large number of factors [20], in the case of sedative muscle relaxation and sufficient oxygen supply, each respiratory motility is relatively constant, and the pressure in the thoracic and abdominal cavity does not change much, so the diagnostic value is still possible. And the degree of inferior vena cava variability is linearly related to arterial lactic acid and can also be used as a reference indicator for early evaluation of shock. The diagnostic value of CVP is obviously lower, which may be affected by other aspects such as respiratory movement and chest and abdominal pressure. Therefore, it is necessary to be cautious as an observation index. However, whether it is inferior vena cava variability or CVP, because it is greatly affected by abdominal cavity and thoracic pressure, it will still affect the value of inferior vena cava variability and CVP under the condition of large changes in abdominal pressure or chest pressure [21, 22].

## Figures and Tables

**Table 1:** Average and standard deviation (SD) of the shock indexes.

	Control (*n* = 166)	Shock (*n* = 138)
Arterial blood lactate	1.04 ± 0.76	4.05 ± 2.54
Caval index	0.59 ± 3.94	0.54 ± 0.17
Central venous pressure	4.94 ± 2.49	3.85 ± 2.26

**Table 2:** ROC (receiver-operating characteristic) curve analysis of the indexes.

	AUC	Std. error	95% confidence interval	*p* value
Arterial blood lactate	0.93	0.015	0.90–0.96	<0.00^*∗*^
Caval index	0.87	0.02	0.83–0.91	<0.00^*∗*^
Central venous pressure	0.63	0.03	0.57–0.70	<0.00^*∗*^

^*∗*^Statistical significance: *p* < 0.05.

**Table 3:** Correlation analysis.

	Pearson's *r*	95% confidence interval	*p*
Caval index/arterial blood lactate	0.30	0.13 to 0.43	0.00^*∗*^
CVP/arterial blood lactate	0.07	−0.09 to 0.24	0.39

^*∗*^Statistical significance: *p* < 0.05.

## Data Availability

The datasets used in the current study are available from the corresponding author upon reasonable request.
